# [Retracted] RNAi-mediated A disintegrin and metalloproteinase 9 gene silencing inhibits the tumor growth of non-small lung cancer *in vitro* and *in vivo*

**DOI:** 10.3892/mmr.2026.14018

**Published:** 2026-09-14

**Authors:** Liang Chang, Fangchao Gong, Youbin Cui

Mol Med Rep 12: 1197–1204, 2015; DOI: 10.3892/mmr.2015.3477

Following the publication of the above paper, it was originally drawn to the Editor's attention by a concerned reader that certain of the tumor images of the mice shown in Fig. 5A on p. 1202 were strikingly similar to tumors that appeared subsequently in a pair of other papers in different journals written by different authors at different research institutes. While the possibility of repeating the experiments shown in Fig. 5 and publishing a corrigendum for this paper featuring revised data in Fig. 5 was considered, a different reader has contacted the Editorial Office to notify us that the western blot data featured in Fig. 1 on p. 1200 were strikingly similar to data that had already appeared in an article in the journal *BMC Biomedicine* that was also written by different authors at different research institutes.

Upon investigating this matter, the concerns of the reader were proven to be well-founded. Owing to the fact that the contentious data in the above article had already been submitted for publication prior to its submission to *Molecular Medicine Reports*, the Editor has decided that this paper should be retracted from the Journal. The authors were asked for an explanation to account for these concerns, but the Editorial Office did not receive a reply. The Editor apologizes to the readership for any inconvenience caused.

